# Gallstone Ileus in a 25-Year-Old Female With Cyclin-Dependent Kinase-Like 5 Deficiency Disorder: A Case Report

**DOI:** 10.1155/crgm/4901433

**Published:** 2025-03-23

**Authors:** Hikaru Onoda, Mami Kuroda, Ryo Takeguchi, Ryosuke Tanaka, Daisuke Ishii, Hisayuki Miyagi, Masatoshi Hirasawa, Satoru Takahashi

**Affiliations:** ^1^Department of Pediatrics, Asahikawa Medical University, Asahikawa, Japan; ^2^Department of Surgery, Asahikawa Medical University, Asahikawa, Japan

**Keywords:** CDKL5 deficiency disorder, cholelithiasis, gallstone ileus, pneumobilia

## Abstract

Gallstone ileus is an uncommon complication of cholelithiasis and the delayed diagnosis may be associated with increased risk of mortality. When gallstones block the cystic duct, they can lead to cholecystitis. If a fistula forms between the inflamed gallbladder and the adjacent intestine, the gallstones may pass into the intestinal tract and cause obstruction in the intestine. We report a case of 25-year-old female with developmental and epileptic encephalopathy who was intraoperatively diagnosed with gallstone ileus during surgery for small bowel obstruction of unknown origin. The patient had potential risk factors enhancing the formation of cholesterol gallstones, including long-term use of phenobarbital, vagus nerve injury in open gastrostomy and laparoscopic fundoplication, and tube feeding; however, the patient's gallstone had been undiagnosed for a long time. Computed tomography of the abdomen showed small bowel obstruction and pneumobilia. The presence of pneumobilia in a patient without a surgical history of the biliary system should raise suspicion of a bilioenteric fistula. The awareness of this complication of cholelithiasis is important to make an early diagnosis and to initiate the appropriate treatment.

## 1. Introduction

Gallstone ileus is an uncommon complication of cholelithiasis, occurring in 0.3%–0.5% of patients with gallstones [1]. However, it occurs more commonly in the elderly, accounting for 25% of nonstrangulated small bowel obstructions in adults over the age of 65 [2]. In a single hospital experience, age of 40 patients with gallstone ileus ranged from 43 to 92 years, with a median age of 78.3 years [3]. When gallstones block the cystic duct, they can lead to cholecystitis. If a fistula forms between the inflamed gallbladder and the adjacent intestine, the gallstones may pass into the intestinal tract and cause obstruction in the intestine [1]. Patients present with nonspecific symptoms such as intermittent abdominal pain, distension, nausea, vomiting, and constipation as the gallstone travels through the gastrointestinal tract. Thus, the diagnosis is often delayed, leading to high mortality rates [2]. Here, we report a case of a young female who was intraoperatively diagnosed with gallstone ileus during surgery for small bowel obstruction of unknown origin.

## 2. Case Report

A 25-year-old Japanese female presented to our pediatric department with fever and vomiting. The patient had suffered from cyclin-dependent kinase-like 5 (CDKL5) deficiency disorder, a severe developmental and epileptic encephalopathy characterized by early-onset pharmacoresistant seizures and a profound delay in psychomotor development. She developed the first infantile spasms at 4 months, followed by generalized tonic seizures. Her seizures were unresponsive to antiseizure medications including phenobarbital, clobazam, zonisamide, and lacosamide. At the time of admission, the patient was receiving phenobarbital at a dose of 8 mg/kg/day, with a blood concentration of 49 μg/mL. Her psychomotor development was severely delayed; she was confined to bed for long periods. She was fed via a gastrostomy tube because of feeding problems resulting from swallowing difficulties. Her gastrostomy tube had been placed via open surgery at the age of 9 years because of pronounced epigastric anatomical abnormality resulting from severe scoliosis. In addition, to treat gastroesophageal reflux disease, laparoscopic fundoplication was performed without vagal nerve preservation.

**Figure 1:**
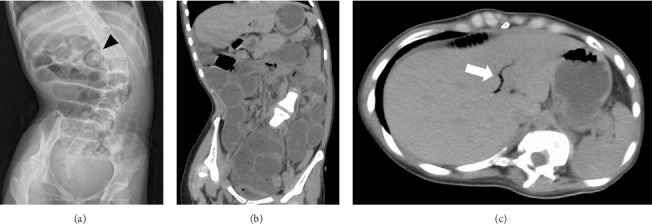
Plain radiograph and computed tomography (CT) of the abdomen in a patient with gallstone ileus. Abdominal radiograph demonstrates dilated small bowel (a). A gastrosomy button is indicated by arrowhead. Coronal (b) and axial (c) CT images demonstrate the small bowel obstruction and pneumobilia (arrow), respectively.

On admission, physical examination revealed abdominal distension but no acute abdomen signs such as rigid abdomen, guarding of the abdomen, and rebound tenderness. Blood test results showed leukocytosis (12,850 cells/μL) and slightly elevated *C*-reactive protein levels (0.53 mg/dL). Chemical workup was normal, except for a slight elevation in the levels of *γ*-GTP (67 U/L) and total cholesterol (229 mg/dL). The total cholesterol level on admission was slightly elevated most likely due to postprandial effects because the high value did not persist after admission. An abdominal x-ray revealed marked dilatation of the small bowel (Figure 1(a)). We considered gastroenteritis or adhesive intestinal obstruction since the patient had a history of open abdominal operation for gastrostomy placement. The patient was treated with fasting and intravenous rehydration but did not improve. On the fourth day after hospitalization, computed tomography (CT) of the abdomen was taken because small bowel obstruction was suspected. It showed small bowel obstruction and pneumobilia (Figures 1(b) and 1(c)) but could not detect mass in the intestine. Although the presence of pneumobilia in a patient without a surgical history of the biliary system should raise suspicion of a bilioenteric fistula, the cause of the obstruction remained unclear at this stage.

**Figure 2:**
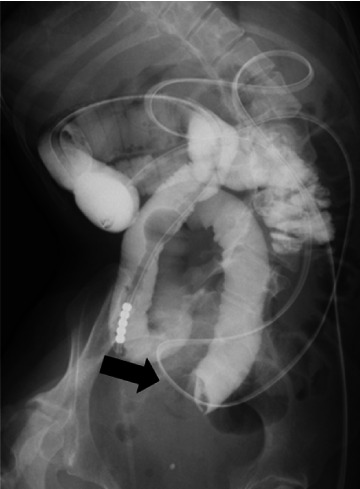
Contrast radiology using an ileus tube. Contrast radiology revealed intestinal retention of the contrast medium and a circular shadow in the intestinal cavity (arrow), measuring approximately 2.5 cm in diameter.

**Figure 3:**
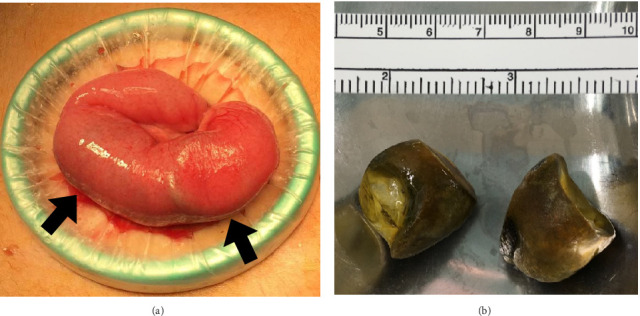
Intraoperative photographs. Large round masses (arrows) were found in the jejunum (a). The extracted gallstones measured approximately 2.5 × 2.5 cm and 3 × 2.5 cm (b).

She underwent an ileus tube insertion via the gastric fistula to decompress the bowels, but her symptoms were not relieved. On the 11th day after hospitalization, contrast radiology through the ileus tube revealed bowel retention of the contrast medium and a circular shadow within the bowel cavity (Figure 2). On the 19th day after hospitalization, surgical treatment was decided, and the initial laparoscopic inspection of the entire small and large bowels detected a mass impacted in the jejunum (Figure 3(a)). An enterotomy was performed over the mass, and two gallstones were extracted, measuring approximately 2.5 × 3.5 and 3.0 × 2.5 cm, respectively (Figure 3(b)). Abdominal ultrasound had never been performed preoperatively.

The patient was diagnosed with gallstone ileus intraoperatively; however, severe adhesions around the gallbladder precluded identification of the bilioenteric fistula. Pathological examinations revealed that the stones contained 98% cholesterol. The postoperative course was uneventful. The patient was discharged from the hospital on the seventh postoperative day and remained asymptomatic after a follow-up of 2 years. Phenobarbital was discontinued and replaced by another antiseizure medication sodium valproate. During follow-up, a magnetic resonance cholangiopancreatography was performed and revealed a gallbladder-duodenal fistula and a small stone in the common bile duct. However, ultrasound could not detect the fistula and the stone because of a large amount of intestinal gas.

## 3. Discussion

We describe a case of a young female with gallstone ileus which was not diagnosed until the surgery on the 19th day after hospitalization. The diagnostic delay in our patient might be partly due to the radiolucent gallstones and a lack of a history of biliary diseases. Previous studies reported that the radiological findings specific to gallstone ileus included mechanical obstruction of the small bowel, pneumobilia, and an ectopic gallstone within the bowel lumen [4]. However, the radiolucent gallstone may be missed. Therefore, the presence of pneumobilia in a patient without a surgical history of the biliary system should raise suspicion of a bilioenteric fistula.

Our patient's gallstone had been undiagnosed for a long time, which might be associated with the diagnostic delay. The mechanisms behind the formation of cholesterol gallstones are not fully understood, but several pathogenic factors are proposed, including genetic background, hepatic hypersecretion of cholesterol, gallbladder dysmotility, and intestinal factors such as increased absorption of cholesterol [5]. In genetically modified mice, lack of cholecystokinin (CCK), a gastrointestinal hormone that can induce gallbladder contraction, induced gallbladder hypomotility that prolonged the residence time of excess cholesterol in the gallbladder, leading to rapid crystallization and precipitation of solid cholesterol crystals [6]. Thus, gallbladder dysmotility seems to be a key factor that triggers the precipitation of cholesterol microcrystals from supersaturated lithogenic bile [5, 7, 8]. It is noteworthy that injury to the vagus nerve has been proposed to be associated with occurrence of gallstones after gastrectomy [9]. Laparoscopic treatment for gastroesophageal reflux disease also poses potential risk of injury to the vagus nerve [10]. Vagus nerve stimulation promotes the motilities of gallbladder and Oddi's sphincter to excrete bile [11], whereas the vagus nerve injury causes prolongation in the gallbladder empty time [12]. Our patient had a history of open gastrostomy and laparoscopic fundoplication without vagal nerve preservation, which might be the potential cause of gallstone formation. In addition, estrogen, a sex hormone that plays an important role in female growth and reproductive development, promotes hepatic secretion of biliary cholesterol and impairs gallbladder motility function [13]. This may contribute to a higher prevalence of gallstones in females than in males. Our female patient had at least two additional risk factors enhancing the formation of cholesterol gallstones: long-term use of phenobarbital and tube feeding via a gastrostomy tube. The patient had been treated with phenobarbital since the age of 9 years. Repeated phenobarbital treatments induce cholesterogenesis in the liver, which contributes to elevated serum cholesterol levels. Previous studies have provided evidence that serum cholesterol levels were increased in children with epilepsy receiving long-term phenobarbital therapy [14]. In addition, bedridden individuals may be at risk of gallstone formation, depending on the feeding methods. Total parenteral nutrition and enteral tube feeding impair gallbladder contraction by decreasing the secretion of CCK and are involved in the formation of gallstones [15].

Thus, our patient had a risk of cholelithiasis that was never recognized. The potential causes of gallstone formation include vagus nerve injury in open gastrostomy and laparoscopic fundoplication and tube feeding that might cause gallbladder dysmotility, and long-term use of phenobarbital that might cause hepatic hypersecretion of cholesterol. To reduce the potential risk of recurrence, phenobarbital was discontinued and replaced by another antiseizure medication. Although the bilioenteric fistula may naturally close without surgery, the patient could still be at risk of recurrence, retrograde cholecystitis, and gallbladder cancer. Therefore, long-term monitoring is essential.

In conclusion, gallstone ileus must be considered in intestinal obstructions, even in younger individuals with the potential risk of cholelithiasis. The awareness of this complication of cholelithiasis is important to make an early diagnosis and to initiate the appropriate treatment.

## Data Availability

The data used to support the findings of this study are available from the corresponding author upon reasonable request.
